# End-of-Life Cancer Care Resource Utilisation in Rural Versus Urban Settings: A Systematic Review

**DOI:** 10.3390/ijerph17144955

**Published:** 2020-07-09

**Authors:** Jessica Cerni, Joel Rhee, Hassan Hosseinzadeh

**Affiliations:** 1 School of Health and Society, Faculty of Social Sciences, University of Wollongong, Wollongong, NSW 2522, Australia; hassanh@uow.edu.au; 2 General Practice Academic Unit, School of Medicine, Faculty of Science, Medicine and Health, University of Wollongong, Wollongong, NSW 2522, Australia; jrhee@uow.edu.au; 3 Illawarra Southern Practice Based Research Network (ISPRN), University of Wollongong, Wollongong, NSW 2522, Australia; 4 Centre for Positive Ageing + Care, HammondCare, Hammondville, NSW 2170, Australia

**Keywords:** end-of-life care, palliative care, cancer care, urban-rural, healthcare service

## Abstract

Background: Despite the advances in End-of-life (EOL) cancer care, disparities remain in the accessibility and utilisation of EOL cancer care resources. Often explained by socio-demographic factors, geographic variation exists in the availability and provision of EOL cancer care services among EOL cancer decedents across urban versus rural settings. This systematic review aims to synthesise mortality follow-back studies on the patterns of EOL cancer care resource use for adults (>18 years) during end-of-life cancer care. Methods: Five databases were searched and data analysed using Preferred Reporting Items for Systematic Reviews and Meta-Analyses guidelines. Inclusion criteria involved; a) original research; b) quantitative studies; c) English language; d) palliative care related service use in adults (>18 years) with any malignancy excluding non-melanoma skin cancers; e) exclusive end of life focus; f) urban-rural focus. Narrative reviews and discussions were excluded. Results: 24 studies met the inclusion criteria. End-of-life cancer care service utilisation patterns varied by rurality and treatment intent. Rurality was strongly associated with higher rates of Emergency Department (ED) visits and hospitalisations and lower rates of hospice care. The largest inequities between urban and rural health service utilisation patterns were explained by individual level factors including age, gender, proximity to service and survival time from cancer diagnosis. Conclusions: Rurality is an important predictor for poorer outcomes in end-of-life cancer care. Findings suggest that addressing the disparities in the urban-rural continuum is critical for efficient and equitable palliative cancer care. Further research is needed to understand barriers to service access and usage to achieve optimal EOL care for all cancer patient populations.

## 1. Introduction

### Background

Worldwide, the demand for end-of-life (EOL) care is growing with the global estimated number of adults in need of EOL care over 19 million [1]. The majority of this demand is due to the increasing prevalence of serious illnesses such as cancer [2]. EOL care continues to provide significant benefits to cancer decedents from relieving symptoms and pain, improving quality of life, and in some cases, survival [3]. Despite these benefits, disparities remain in access to and utilisation of EOL cancer care resources often influenced by a variety of factors from government investment, service delivery practices and socio-demographic factors, particularly among those in rural communities compared with urban areas [4].

Several pervasive social and structural factors have been identified as being important determinants of the patterns of EOL cancer care including barriers at the patient, physician and healthcare system level [5]. Previous research has shown that geographic variation exists in the availability and provision of EOL cancer care services within and between countries [6], particularly among vulnerable groups including the elderly and cancer decedents living in rural settings. Access to such care for rural and remote patients and carers has been identified as an area in particular need of focused research [7]. Geographic remoteness or rurality in the health context is defined as living outside of places with population densities of 400 or more per square kilometre [8]. This is of primary interest, given the extent of inequities indicating rurality to be a strong predictor of high utilisation rates of EOL acute-care [9,10,11,12,13] and community based care services [13,14] and disproportionately low access and underutilisation of palliative care services [15,16,17,18].

The underlying reasons why EOL cancer care is often disproportionately underutilised, provided ineffectively or not provided at all among patients residing in rural and regional areas [19] is complex and often explained by socioeconomic and geographical factors such as resource and funding restraints in rural areas [20]. Previous reviews have highlighted EOL care to be an essential service in rural communities, yet the literature also suggests there is a relationship between rurality and poorer quality of EOL care [21]. Given the complexity in defining rurality, there remains a clear need to examine the influence of rurality and patterns of healthcare service utilisation by EOL cancer patients [21]. After a thorough examination of all past and relevant reviews, including those in the Cochrane Library database, it is clear that no study has systematically reviewed the literature that examined the association between rurality, in particular urban versus rural region of residence, and the utilisation patterns of healthcare services in EOL cancer decedents.

Providing equitable EOL healthcare to individuals, irrespective of their region of residence presents as a significant challenge to healthcare systems worldwide. Exploring the types and patterns of healthcare services used among underserved populations such as rural residents during their last months of life is crucial to understanding the quality and appropriateness of care provided. Research using secondary health administrative data can provide crucial insight into the treatment patterns of urban and rural residents enduring EOL cancer care. The findings uncovered by these large datasets have the potential to inform policies that are tailored to addressing the barriers faced by vulnerable populations such as rural residents in order to respond to the challenge by enabling universal access to quality EOL cancer care [2]. This systematic review provides a clear outline of the methodology used to find relevant studies exploring rurality and EOL cancer care along with a comprehensive analysis of the interplay between these factors in the results and discussion sections.

In addition to the strong evidence on the benefits of EOL cancer care in relieving symptoms and pain, improving quality of life, and in some cases, survival (3), there is also evidence that high quality palliative care could lead to reduced hospitalisations and emergency department (ED) presentations [22]. The challenge lies in ensuring equity in the delivery of EOL cancer care across all populations, particularly those living in rural and remote areas where limited resourcing, across a large geographic area with decreased population density remains a key factor. The aim of this systematic review is to synthesise mortality follow-back studies on the influence of rurality on the patterns of EOL cancer care service use in decedents with cancer.

## 2. Materials and Methods

### 2.1. Selection Protocol

**Figure 1:**
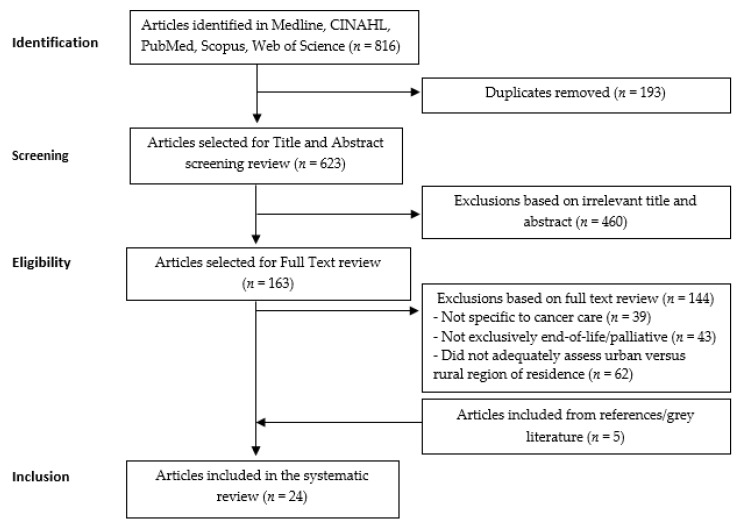
Flowchart of publications included in the systematic review.

The systematic review followed the Preferred Reporting Items for Systematic Reviews and Meta-Analyses guidelines [23]. Study identification and selection process is displayed in Figure 1. Search protocol was independently conducted by the authors with predefined search terms and strategies from databases of published peer-reviewed literature.

### 2.2. Search Strategy

A search was conducted in Medline, The Cumulative Index to Nursing and Allied Health Literature (CINAHL), PubMed, Scopus and Web of Science using keywords “healthcare” OR “health service” OR “hospitalization” OR “health service utilization” AND “cancer” OR “neoplasm” OR “malignant” OR “carcinoma” OR “palliative care” OR “end of life care” OR “terminal care” AND “rural” OR “remote”. No limits applied. Reference lists were scanned to ensure all related publications were included (Supplementary Table S1).

### 2.3. Inclusion/Exclusion Criteria

Studies reporting on health service utilisation at end-of-life occurring during any defined period were included. Articles without a definition of rurality and/or a comparison group (rural versus urban) were excluded. Inclusion criteria included original articles examining urban versus rural residence (e.g., rurality, geographic remoteness, urbanisation, region of residence) of relevance to at least one end-of-life palliative care service utilisation related outcome in adult cancer patients (≥18 years), published post-1990 in the English language. Exclusion criteria included reviews to minimise the risk of including the same studies more than once, discussions and any other non-original articles (Supplementary Table S2).

### 2.4. Data Extraction and Analysis

Relevant data were extracted using a customised Microsoft Excel spreadsheet adapted from the Cochrane Systematic review template [24]. With a random sample selected, all authors’ cross-checked extracted data and any disagreements that arose were resolved through discussion and further analysis. Study quality and risk of bias was assessed using the protocol developed by Dzhambov et al. [25] and the 1.0 point checklist from the National Heart Lung and Blood Institute (NHLBI) Quality Assessment Tool for Observational Cohort and Cross-sectional Studies [26]. Based on a set of 14 criteria, a quality rating was agreed upon by the authors using a score of Yes = 1, No = 0, Cannot Determine = 0 in response to each question. Answers of Not applicable = 0 were excluded from the total applicable count. A quality score was calculated as a percentage calculation of (total positive/total applicable) and classified as: <0.50 (low quality), 0.50–0.74 (fair quality) and 0.75–1.00 (high quality). Results were discussed and any discrepancies were resolved by the authors. The checklist included 14 questions with 12 applicable to observational mortality follow-up study designs (Supplementary Table S3). The criteria assessed the research question, study population, population eligibility criteria, sample size justification, exposure measures and assessment, study timeframe, outcome measures, follow-up rate and statistical analyses.

Owing to clinical and methodological heterogeneity of identified studies it was not possible to conduct a meta-analysis, thus, a narrative synthesis of quantitative results was conducted. The key study characteristics of the included studies are summarised, including the results in (Supplementary Table S4).

## 3. Results

In total, 816 studies were identified through five databases using selected search terms (Supplementary Table S1). Of these, 653 studies were removed based on title and abstract review with 193 being duplicates (Figure 1). From the remaining 163 studies, 144 studies were excluded after full text review as they did not compare urban versus rural outcomes. An additional five studies were identified and included through cited references and google scholar searches.

### 3.1. Characteristics of Included Studies

Twenty-four studies were included in the review (*n* = 100%). All studies employed quantitative methodologies linking pre-existing secondary data to area-level specific residential geography classification data. All studies used retrospective mortality follow-back study designs. The majority of the articles were published in the last decade (58%) and represented health databases of populations in Canada (38%), USA (38%), Taiwan (13%), Australia (8%) and Germany (3%).

### 3.2. End-of-Life Period Classification

All studies measured a defined end-of-life period that ranged from 2 weeks to 18 months before death. Eighteen studies (75%) classified the end-of-life period as less than the last 12 months of life. Three studies (13%) included a shorter time period, that is, the last 2 weeks of life [9,17,27] (Table 1, Supplementary Table S3).

### 3.3. Urban Versus Rural classification

Studies that compared findings from both urban and rural populations were of primary interest. Twenty definitions and measures of rurality were used across the studies (Supplementary Table S5). These included derivations of “region of residency” defining rural areas as communities with populations <10,000 people [28], urban areas as regions with a population density of >400 persons per km^2^ [10] and both rural and urban areas were defined as a non-metropolitan and metropolitan county [17], respectively. A range of global databases were used to measure each exposure variable including: postal code data, Surveillance, Epidemiology and End Results Medicare data registry (SEER) database and Accessibility Remoteness Index of Australia (ARIA) database classifications. The most common index used to measure rurality was objective measures of population concentration derived from 2006 Canadian Census of the Populations and the Registered Persons Database (RPD). This was assessed in four studies (17%) [9,11,28,29].

### 3.4. Cancer Classification and Tumour Characteristics

Cancer diagnoses were categorised according to the International Classification of Disease (ICD) codes (*n* = 19), SEER [17,18] (*n* = 2) and the Cancer Agency Information System (CAIS) [30] (*n* = 1). Cancer diagnoses were identified using administrative health database claims (*n* = 11), cancer registries (*n* = 10), death certificates [31] (*n* = 1), health insurance claims [32] (*n* = 1), palliative program enrolment records [33] (*n* = 1) and linked cancer registry and death certificate data [34] (*n* = 1). Using the ICD classification codes, 13 types of cancer were assessed (Table 1) with the most common being lung (*n* = 15) and colorectal (*n* = 14). The stage of tumour at diagnosis was assessed in five studies [12,14,17,18,35] using two classification systems; the historic staging system collected using SEER data and the American Joint Committee on Cancer classification system [18]. Studies that assessed the stage of tumour at diagnosis included stages I–IV specific to colorectal cancer [35], stages III–IV specific to advanced breast cancer [17] and stages I–IV specific to lung and colorectal cancer [18].

### 3.5. Healthcare Service Utilisation—Treatment Intent

Nine types of healthcare services were included (Table 1) that reflect three classifications of treatment intent. Life-sustaining treatment includes chemotherapy (*n* = 7), ED visit/s (*n* = 10), hospital admission/s (*n* = 8) and Intensive Care Unit (ICU) admission/s (*n* = 6). Community-based care includes home health, home doctor, physician and general practitioner (GP) visit/s (*n* = 3). Palliative care includes specialist palliative care (SPC) services (*n* = 2) inclusive of hospice services (*n* = 10), palliative radiotherapy (PRT) (*n* = 2) and pharmacotherapy (*n* = 1).

### 3.6. Study Quality

Using the 1.0 point checklist from the NHLBI Quality Assessment Tool for Observational Cohort and Cross-sectional Studies [26], two (8%) studies were deemed high quality having justified sample size, power descriptions or variance and effect estimates. Eighteen studies (75%) were deemed fair quality with many using a pre-specified and uniformly applied inclusion and exclusion criteria and sufficient study timeframe. Four studies (17%) were deemed low quality due to the lack of comprehensive statistical analysis or measurement and adjustment for confounding factors.

### 3.7. Results by Primary Outcome

#### 3.7.1. Urban-Rural Effect on Acute and Life-Sustaining Healthcare Services Use

Seven studies assessed the utilisation of chemotherapy [9,11,12,13,29,35,36], 10 studies assessed the number of ED visits [9,10,11,12,27,28,29,33,35,36], eight assessed hospital admissions [11,12,13,15,29,35,36] and six studies assessed the number of ICU admissions [9,11,12,29,35,36] in relation to rurality (Table 1). Among entire death cohorts who received chemotherapy in the last 14 days of life in a hospital setting, irrespective of rurality, the rates of chemotherapy usage varied from 2–4.5%. When assessed as an independent variable and in relation to rurality, four studies found no difference in the odds of receiving chemotherapy in the last 14 days of life according to rural region of residency (*p* > 0.05) [9,13,29,35].

The rates of ED visits, irrespective of rurality, also varied considerably from 27–60% of death cohorts depending on the period in which as least one ED visit was recorded across a three-year study period, the last six months of life and the last 30 or 14 days of life. When assessed over a three-year study period, one study concluded 0.76 lower ED visits (95% CI, 0.23–2.36) among residents in urban areas than rural even after controlling for out-of-hours periods of palliative care home care [33]. When assessed over the last six months of life, one study found rural region of residence increased the odds of having at least one ED visit, with 5% spending more than 30 days in hospital (OR 1.25, 95% CI, 0.2–1.90) [28]. Another study also found rural residency to increase the rate ratio of the total number of ED visits after controlling for patient characteristics and the effect of continuity of care with a family physician (OR 1.29, 95% CI, 1.21–1.37) [10]. Similar results were found in four studies where higher rates of ED visits were reported in the last 30 days of life [29,35] among cancer decedents living in rural areas compared to urban areas with rates reducing with narrower time periods to death [35] such as the last two weeks of life [9,27].

The rates of more than one hospital admission varied from 8.5–85.4% of death cohorts in their last 30 days [29] and last year of life [16], respectively, significantly reducing with narrower time periods to death. In the last three years prior to death, two studies found decedents with a rural geographic region of residence to have an increased likelihood of having at least one hospital admission [15,16]. Similarly, over the last 30 days of life, two studies also found rural region of residence to have a strong association with multiple hospitalisations [29,35] with Hu et al. [35] reporting cancer decedents of Calgary (rural region) as having 3.3–5.3 times the adjusted odds of being hospitalised more than once. Additionally, Walter et al. [13] found the likelihood of having more than 14 hospital days in the last 30 days of life to be significantly higher for decedents in rural districts than in remote rural districts (1.27 (1.05, 1.52), (*p* = 0.0003).

Three studies examined ICU admissions as a primary outcome. When assessed over the last 2 weeks of life, Hu et al. [35] found the rate of ICU visits among cancer patients in Ontario to be 5.4% of the cohort and rurality to be significantly associated with ICU admissions. When assessed over the last 30 days of life, this rate increased to 7.2% [29]. One study found rural region of residence to be strongly associated with an increase in ICU admissions (1.91 (0.77–4.73), *p* > 0.05) among colorectal cancer patients in Alberta. However, in contrast, Conlon et al. [29] were unable to find similar evidence, finding no association between rural locations and the odds of receiving intensive care in the last 30 days among a cohort of Ontario cancer patients. In multivariable analyses, when the use of chemotherapy, ED visits, hospital and ICU admissions were assessed in a composite measure of aggressive EOL care, three studies found decedents were more likely to receive aggressive EOL care if they resided in rural regions compared to those in urban or metropolitan areas [11,12,36].

#### 3.7.2. Urban–Rural Effect on Community-Based Healthcare Services Use

Three studies explored the association between rurality and GP visits [13] and home doctor services [15,16] (Table 1). In a cohort of lung cancer patients, the number of visits to the GP (physician) in the last 30 days of life was significantly lower in urban districts than in remote rural districts (β = −0.19 (−0.32, −0.06), *p* = <0.0001) [13]. Similar results were also found in a data linkage study of palliative colorectal and lung cancer patients in the United States where those living in a rural geographic region were slightly more likely to use home health services (1.19 (1.02–1.39)) than those in urban regions [15].

#### 3.7.3. Urban-Rural Effect on Palliative Healthcare Services Use

As measures of specialist palliative care two studies examined SPC usage [31,37], 10 studies examined the use of hospice care [12,14,15,16,17,18,32,38,39,40], two studies assessed receipt of PRT [30,34] and one study assessed receipt of pharmacotherapy [13] (Table 1). Regarding SPC usage, Burge et al. [37] found residents in rural regions compared with urban were less likely to be registered with a palliative care program (PCP) after controlling for age (0.8 (0.7–1.0)). Likewise, Rosenwax and McNamara [31] also found a disproportionate distribution of SPC usage by rurality finding cancer decedents were significantly less likely to receive SPC in the last 12 months of life if they lived in a region other than a major city.

The rates of hospice enrolment and utilisation, irrespective of rurality, varied considerably across the studies from 7% enrolment at more than 180 days before death [18] to 63% of the death cohort enrolled more than 7 days before death [38]. Despite an increasing trend of hospice demand and use in rural areas over the study periods in two studies [14,32], a consistent finding was that the odds of hospice enrolment was lower for patients residing in a rural area than an urban or metropolitan region [14,15,16,17,18,32,38,39,40]. Additionally, when assessed as a composite measure of potentially aggressive EOL care within the last 30 days, including (chemotherapy received within 14 days of death, >1 emergency department (ED) visit within 30 days of death, >1 hospitalisation within 30 days of death, ≥1 intensive care unit (ICU) admission within 30 days of death, in-hospital death and hospice enrolment ≤3 days before death) decedents who resided in non-metropolitan areas were more likely to have aggressive care including late and short hospice enrolment (less than 3 days before death) [12].

Assessed in just two studies, the patterns of PRT treatment rates were observed to be low, geographically-dependent and vary from 14% at any point after prostate cancer diagnosis [30] to 22.5% in the last 9 months of life in decedents with lung, breast, melanoma and prostate cancer [34]. Soo et al. [30] found more remote regions to have lower PRT utilisation rates and decedents who resided in areas that were geographically removed from a cancer centre were less likely to receive PRT. Similarly, Lavergne et al. [34] also found, through univariate analysis that living in a rural area was a predictor of lower rates of PRT consultation and treatment. In a multivariate analysis however, rural residence was no longer significant due to collinearity with travel time and deprivation [34]. Just one study examined the urban-rural differences in EOL patterns of pharmacotherapy with antidepressants and pain relief medication [13]. Examining the receipt of pharmacotherapy treatment with antidepressants and structured pain relief during the last 30 days of life, Walter et al. [13] were unable to find any regional differences in pharmacotherapy treatment patterns.

The interpretation of these findings is challenging because of the heterogeneity of methodologies, classification of study periods and rurality. It seems clear, however, that decedents residing in rural or remote areas had higher odds of receiving more acute-aggressive care and community-based care and lower odds of receiving palliative care including SPC services such as hospice and palliative radiotherapy.

#### 3.7.4. Covariate Effect

Twenty-two studies assessed effect estimates of individual and geographical area level covariates including age, gender, marital status, insurance, cancer diagnosis and stage and distance to health service (Table 1). Specific to the use of acute and life-sustaining healthcare services, being younger (<60 years), having breast, lung, or hematologic malignancies, having a rural postcode, having two or more comorbidities or being male was prognostic for greater odds of an ED visit or death in acute care [11,28]. Having a follow-up visit with an oncologist was also associated with multiple hospitalisations in the last 30 days of life [35]. Being aged (<50 years), male and with a rural region of residence was associated with lower rates of ICU admissions in the last 30 days of life [9,29]. Patients with breast cancer were 88% more likely to receive chemotherapy in the last two weeks of life than patients with colorectal cancer (*p* < 0.001) [11].

The effects of covariates on service usage with community-based care intent, including comorbidities at time of diagnosis (e.g., congestive heart failure, chronic obstructive pulmonary disease) were found to be significantly associated with a lower number of doctor visits [13]. In relation to palliative care services, the location and supply of hospices were found to play a major role in hospice utilisation rates in rural areas [32]. Distance to the closest cancer centre was an important factor in Palliative Care Programs (PCP) use where individuals aged 85 years and over were 17 times more likely to be registered with a PCP if they lived 10 km or less from the cancer centre compared with those who lived over 50 km away [37]. Increasing household income [38], increasing age (65+ years) [17], not married [18,31], being male [18] and having fee-for-service insurance [18] were indicative of lower odds of receiving hospice care and SPC services. PRT treatment rates in the last nine months of life also varied by age and region of residence and declined with increasing travel time to nearest PRT centre [34].

## 4. Discussion

This systematic review of retrospective mortality follow-back design studies provides evidence of higher odds of EOL cancer care acute-care service usage and underutilisation of palliative services with greater geographic remoteness. Limited evidence was found for the influence of geographic remoteness on the use of EOL cancer care community-based services.

Evidence on the influence rurality has on the EOL cancer care among adults has grown over the past decade with the majority of studies coming from a select group of high-income countries. Although heterogeneous in nature, the findings across the studies are markedly similar, that is, finding substantial geographical variation in EOL cancer care. The resource utilisation patterns detailed in this review have important implications particularly for identifying areas in need of further research such as studying patient demographics as predictors of palliative and hospice service utilisation and assessing the appropriateness, timeliness and quality of EOL cancer care by treatment intent with a focus in the rural setting.

### 4.1. Relevance to Prior Knowledge

Unsurprisingly, the majority of the findings of this review remain consistent with the findings of prior reviews where authors also reported an inverse relationship between EOL resource use and age and a consistent trend of underutilisation of palliative services among rural EOL cancer decedents [41]. Past reviews that focussed on healthcare provider and patient barriers and quality of EOL care also identified low provider comfort, limited scope of practice [42] as well as family member’s avoidance issues around dying [43] as being distinct challenges faced by rural communities when it comes to EOL cancer care. From an ecological perspective, this systematic review remains the first to provide additional insight into the influence geographical remoteness has on the patterns of EOL cancer care among patient populations residing in rural and urban locations. With significant variations by geographic remoteness and treatment intent, the patterns of EOL cancer care among rural decedents reflected an over reliance on acute-care services including ED visits [9,10] and hospital admissions in the last 30 days before death [11,12,13] and an underutilisation of palliative services such as hospice care in the last 12 months of life [14,18,31,32,38,40].

The results of this review also highlight the influence confounding factors have in the patterns of EOL cancer care utilisation, namely age [9,10,11,12,13,14,15,16,17,18] and gender [2,3,4,5,6,7,8,9,10,11,12,13,14,15,16,17,18,19,20,21,22,23,24,25,26,27,28,29,30,31,32,33,34,35,36,37,38,39,40], cancer characteristics (stage of diagnosis [17,18,35,40], cancer type [9,10,11,12,13,14,15,16,17,18,27,28,29,30,31,32,33,34,35,36,37,38,39,40], survival time [10,11,13,15,16,29,34,35,36,37,39], comorbidities [9,11,13,14,15,16,17,28,35] and distance to nearest cancer centre [37]. The interplay of these factors is complex as many communities and individuals have overlapping characteristics [37]. This review highlights the current evidence gap that remains in quantifying the theorised pathways of mediation and effect modification to better explain the variation in EOL cancer care provision. Recent research suggests that whilst there is no simple explanation for the growing inequity in EOL cancer care provision across urban-rural populations, referral patterns to specialist palliative care, proximity to services [44], including travel time and road network factors, and socio-economic status may be additional confounding factors worthy of further investigation.

Consistent with the findings of this systematic review, Gao et al. [45] found living more than 30 kilometres from a PCP was associated with lower PCP enrolment. A lack of resources to refer to and reluctance to refer in rural areas is also one of a few reasons postulated by researchers such as Hawley [46] to explain the disparities seen in EOL cancer care across urban and rural settings. Ensuring future studies simultaneously adjust for multiple demographic, health service and socio-cultural indicators will help to provide greater insight into understanding the important determinants of the patterns of EOL cancer care and reviewing public health policies to ensure equity in the distribution and usage of palliative care services.

With the concept of ‘rurality’ having been widely acknowledged in public health policy making and resource allocation [47], a variety of quantitative and qualitative methods have been proposed to define ‘rurality’ for various purposes. These measures are based largely on classifications related to geography, including administrative units such as counties, census tracts or postal-code areas [48]. Across the included studies, 20 different definitions and measures of ‘rurality’ were used, highlighting a high degree of heterogeneity brought about by a lack of standardised metrics to clearly define and measure ‘rurality’ in the context of assessing EOL cancer care on a global scale.

Researchers examining health service access have acknowledged the limitations associated with oversimplifying the definition of ‘rurality’ where significant cultural, demographic and socioeconomic characteristics are commonly overlooked [49]. Nonetheless, throughout the identified studies, definitions that measured both population density and used zip codes to measure distance to urban regions appeared to be a more valid measure of rurality [9,28,29,37]. These criteria attempt to designate ‘rural’ areas beyond a geographic boundary considering other factors such as access to health services and supply of oncology and palliative care services, where the supply of these services is commonly influenced by population demographics [50]. The use of standardised composite indexes to define and measure rurality may be beneficial in future rural health research. Solely focussing public health programs to improve EOL cancer care provision in rural areas on geographic boundary evidence may hinder their effectiveness by diverting attention away from other more fundamental social and structural processes.

Furthermore, eight types of EOL cancer care services were examined in the context of five countries across the studies. Firstly, it is important to note the effects of differing health systems according to the country referred to in the cited studies. According to the country of the studies, one notable difference in the findings was identified in the study by Walter et al. [13], where receipt of antidepressants and pain relief medication were not associated with geographical or regional differences. Noting that this outcome was assessed in just one study, the authors state that regional differences in EOL care are not an issue in a German setting as EOL care providers such as GPs and hospice services are found in every district type [13]. It is therefore plausible that the nature of the healthcare system in their governance, overall design and function, vastness of the geography and cultural ideologies of different countries may be influencing the types of services used, timing of receipt of quality care and patient preferences. Examined in just three studies, our results also indicate that healthcare supply factors such as hospice supply, bed supply and SNF supply may not play as much of an important role in EOL cancer care as location of services. Future research is needed to examine this relationship and others including palliative care delivery programs in other settings.

### 4.2. Strengths and Limitations

The study generated important insights about the impact of rurality on healthcare service utilisation at EOL in people with cancer. The strengths of the study include the comprehensive database search, rigorous quality assessment and assessment of multiple outcome measures covering services with acute care and life sustaining care, community-based care and palliative care intent.

Limitations include the language restrictions of the database search strategy, excluding non-English articles, and thus, potentially resulting in an underrepresentation of research from non-English speaking countries. The issue of residential self-selection across urban and rural geographies may make inferences regarding causality more difficult as patterns of EOL cancer care utilisation based on individual behaviours may appear as an inflated geographic related effect. The methodology of grouping acute-care health services could be problematic, given that for some rural settings it is possible that this represents one of only a few options to get healthcare, especially after-hours. The study results could be influenced from reverse causation bias if these factors are overlooked. The variation in sample sizes and design of the studies and incomplete adjustment of potential predictors involved in explaining the patterns of EOL cancer care by rurality could have also biased the results in some of the studies. Considering the effects of additional infrastructure and healthcare system factors (e.g., hospice supply, physician supply, referral rates, pharmacotherapy) and lifestyle factors (e.g., support networks) may provide a more accurate and valid reflection of EOL cancer care and service utilisation.

Many studies only included cohorts with eligibility for fee-for-service Medicare under the respective national healthcare systems, reducing the generalisability to other populations such as non-insured populations [16] and those in Health Maintenance Organisation plans. Considering the types and levels of insurance cover as a confounding factor in future research may also help to uncover other factors such as financial mechanisms impeding on EOL cancer care treatment patterns [51]. The lack of a standardised approach to defining and measuring rurality also results in difficulty interpreting comparisons deriving from different geographical based burdens and EOL cancer care treatment patterns.

### 4.3. Implications for Practice

While it is evident that rurality plays an important role in the disparities seen in EOL cancer care provision, it does not mean that rurality necessarily leads to these rural-urban disparities. The physical barriers associated with rurality may simply be exacerbating the effects of other potential determinants influencing the availability, accessibility and utilisation patterns of EOL cancer care such as socioeconomic disadvantage, psychosocial health and transport options. Programs to improve the provision of EOL cancer care across urban and rural settings that are multidisciplinary in nature will be most effective as more risk determinants can be addressed. Further research is needed to understand these determinates of selective EOL treatments to improve rural health outcomes and ensure quality palliative care is provided to all EOL cancer patients irrespective of geographical location.

### 4.4. Implications for Science

A growing body of literature and previous reviews suggest that the use of palliative care is associated with improved quality of life and a reduced likelihood of aggressive care [9,21]. In interpreting the results of this review, reconsidering the use of acute-care services such as ED visits as a marker of inappropriate or life-sustaining care when thinking about rural contexts may be required. The finding regarding rurality to be a strong predictor of disproportionately low access and utilisation of palliative care services [15,16,17,18] was not surprising as noted in past research, the underutilisation of palliative services in rural areas may be a reflection of the limited availability of hospice services [52] or slower dissemination of palliative care services or advances [1] to rural communities. As highlighted in a study demonstrating the major role of location and supply of hospices play in hospice utilisation rates in rural areas [32], patients may be presenting to an ED and utilising acute hospitals as a substitute for non-existent palliative care services. Interestingly, Craigs et al. [53] found that patients were less likely to receive palliative care if they had not received an opioid prescription or anti-cancer treatment. Given that the results of this systematic review could not support such a finding, rather than looking at acute-care services or provision of palliative care services, examining receipt of pharmacotherapy and other process measures may help to further evaluate the quality of EOL cancer care and determine the extent of the true disparity in EOL cancer care across urban and rural settings.

### 4.5. Future Research Directions

To better understand the barriers to EOL cancer care service availability, access and usage to achieve optimal palliative care for all patient populations across the urban-rural continuum, further research is needed to calculate the percentage of ED visits and hospital admissions across urban and rural settings that are for palliative care reasons. This could be achieved by looking at discharges from hospice to inpatient setting or hospital admissions coded as inpatient palliative care.

Studies need to employ reliable and valid quantitative methodologies that link pre-existing secondary health data to area-level specific geographical classification data using logistic and multilevel regression analysis techniques. There appears a clear need for a systematic approach to further explore pathways linking geographical factors. Adjusting for infrastructure factors such as hospice availability and referral rates as well as individual level factors such as age, gender, cancer characteristics and SES may help to uncover important determinants to EOL cancer care service use outcomes. Rather than relying on variable geographical measurements of rurality that do not account for country to country differences, including measurements of distance and travel time to the nearest cancer centre or hospice may also help to improve the reliability and validity of future studies.

## 5. Conclusions

Overall, the current evidence suggests rurality is an important predictor for poorer outcomes in the provision of EOL cancer care, especially with the use of services involving palliative care intent. This review highlights the potential of addressing barriers brought by geographical remoteness has in reducing EOL cancer care disparities across urban and rural settings. The data show rurality to be associated with an overreliance on EOL acute-care services and underutilisation of services with palliative care intent. These results highlight the influence rurality has on the patterns of EOL cancer care service utilisation that varied significantly based on the intent of the provided care. Further studies focused on developing a more thorough understanding of the pathways involved in the influence of geographical barriers on EOL cancer care would be useful to help to inform the development of health policies to improve supply, access and utilisation of EOL cancer care services. Thus, responding to the ongoing challenge to provide consistent and reliable access to appropriate and quality EOL cancer care across urban and rural settings.

## Figures and Tables

**Table 1:** Overview of included studies (*n* = 24).

Study Characteristic	Group	Number of Studies ^i^
Year of publication	1998–2004	4
	2005–2012	10
	2013–2019	10
Study setting (country)	Canada	9
	USA	9
	Taiwan	3
	Australia	2
	Germany	1
Study design	Case Control study (CCS)	1
	Cross-sectional study (CSS)	2
	Cohort/Longitudinal study (CS)	21
Statistical analysis methods	Linear Regression	5
	Univariate logistic regression	3
	Binary logistic regression	4
	Multivariate logistic regression	15
	Negative binomial regression	1
	Hierarchical non-linear regression	1
	Cos proportional hazards regression	1
	Poisson Regression	1
	No comprehensive analysis techniques used	1
Quality assessment ^iii^	High	14
	Fair	7
	Low	3
Inclusion criteria (minimum age)	Any age	6
	18–20	9
	65–70	7
	Not Reported	2
End-of-life period	≤1 month	9
	6 months	3
	9 months	1
	12 months	4
	>12 months	1
	Variable ^ii^	2
	Not Reported	4
Cancer types	Lung	15
	Colorectal	14
	Prostate	12
	Breast	11
	Haematological	9
	Gynaecological	4
	Pancreatic	4
	Head and Neck	4
	Upper gastrointestinal	4
	Liver	4
	Melanoma	2
	Central Nervous System	2
Healthcare services	Chemotherapy	7
	ED/ER visit	10
	Hospital admission	8
	ICU admission	6
	SPC service/s	15
	Palliative Radiotherapy	2
	Home doctor/physician visit/s	3
	Prescription medication/s	1

Note: ^i^—includes a cumulative count reflective of studies assessing multiple outcome variables; ^ii^—study quality was assessed using the Quality Assessment Tool for Observational Cohort and Cross-sectional Studies [26], ED—Emergency department; ER—Emergency Room; ICU—Intensive Care Unit, SPC—Specialist Palliative Care, ^iii^—variable EOL period including time from an event or treatment with palliative intent until death, such as palliative radiotherapy, hospice enrolment/use.

## Supplementary Materials

The following are available online at https://www.mdpi.com/1660-4601/17/14/4955/s1, Table S1: Electronic search strategy, Table S2: Eligibility criteria, Table S3: NHLBI Quality Assessment Summary of Results, Table S4: Characteristics summary of included studies, Table S5: Summary of geographical urban-rural measures and influence on end-of-life cancer care health service use outcomes.
