# Supplementary material for: End-of-Life Cancer Care Resource Utilisation in Rural Versus Urban Settings: A Systematic Review
Source: Int J Environ Res Public Health. 2020 Jul 9;17(14):4955. doi: 10.3390/ijerph17144955 (PMC7400508; doi:10.3390/ijerph17144955)
Supplement: Supplementary file 1 [file ijerph-17-04955-s001.pdf]

Table S1. Electronic search strategy.

Database	Search Terms	Records Identified	Date last searched
Scopus	(TITLE-ABS-KEY (healthcare OR "health service" OR hospitalization OR "health service utilization") AND TITLE-ABS-KEY (cancer OR neoplasm OR malignant OR carcinoma OR adenocarcinoma) AND TITLE-ABS-KEY (palliative AND care OR "end of life" OR terminal OR dying) AND TITLE-ABS-KEY (rural OR remote)) AND (LIMIT-TO (DOCTYPE , "ar")) AND (LIMIT-TO (LANGUAGE , "English")) =	153	26/02/20
PubMed	(healthcare [Title/Abstract] OR health service[Title/Abstract] OR hospitalisation [Title/Abstract])) AND (cancer*[Title/Abstract] OR neoplasm*[Title/Abstract] OR metasta* [Title/Abstract] OR malignant[Title/Abstract] OR carcinoma*[Title/Abstract] OR adenocarcinoma [Title/Abstract])) AND (palliative care [Title/Abstract] OR end of life care[Title/Abstract] OR terminal care[Title/Abstract] OR dying [Title/Abstract])) AND (rural*[Title/Abstract] OR remote[Title/Abstract] OR isolated[Title/Abstract] OR regional[Title/Abstract] OR small town[Title/Abstract]))))	69	26/02/20
CINAHL	AB (healthcare OR "health service" OR hospitalization OR "health service utilization") AND AB (cancer* or neoplasm* or metasta* or malignant or carcinoma* or adenocarcinoma*) AND AB (palliative care or end of life care or terminal care or dying) AND AB (rural* or remote or isolated or regional or small town)	93	26/02/20
Medline	AB (healthcare OR "health service" OR hospitalization OR "health service utilization") AND AB (cancer* or neoplasm* or metasta* or malignant or carcinoma* or adenocarcinoma*) AND AB (palliative care or end of life care or terminal care or dying) AND AB (rural* or remote or isolated or regional or small town)	90	26/02/20
Web of Science	TS=(healthcare OR health service OR hospitalization OR health service utilization) AND TS=(cancer* OR neoplasm* OR metasta* OR malignant OR carcinoma* OR adenocarcinoma*) AND TS=(palliative care OR end of life care OR terminal care OR dying) AND TS=(rural* OR remote OR isolated OR regional OR small town)	411	26/02/20

Table S2. Eligibility criteria.

**Study Component**	**Inclusion Criteria**	**Exclusion Criteria**
Study Design	Quantitative studies, cross-sectional studies, cohort studies	Review, Discussion, Qualitative studies
Study characteristics	English language<br>Full-length journal article published in peer-reviewed journal<br>Publication date post 1990	Languages other than English
Study population	Individuals (18 years+) with any malignancy excluding non-melanoma skin cancer (cancer patient/s)	Individuals with no cancer diagnosis<br>Cancer patient/s with non-melanoma skin cancer
Data Sources	Healthcare service utilisation exclusively for cancer related palliative and/or end-of-life period as stated by the authors (e.g., hospital admission, prescription dispensation, GP/doctor visits)	Non-cancer related healthcare service use outcomes<br>Cancer related healthcare service use outcomes without reference to end-of-life and/or palliative care
Palliative Care Focus	Exclusive focus on palliative and/or end-of-life period as stated by the authors	Studies examining other stages of cancer care (e.g., diagnosis, post-surgery)
Urban-Rural Focus	Rural versus urban residential focus of palliative and/or end-of-life care	Palliative and/or end-of-life cancer care without an urban-rural focus

Table S3. NHLBI Quality Assessment Summary of Results.

Author, Year	1	2	3	4	5	6	7	8	9	10	11	12	13	14	TOTAL POSITIVE	TOTAL APPLICABLE	%	RANKING
Bainbridge et al., 2015	Y	Y	Y	Y	N	N	Y	NA	Y	N	Y	NA	CD	Y	8	12	0.67	FAIR
Barbera et al., 2006	Y	Y	Y	Y	N	N	Y	NA	Y	N	Y	NA	CD	Y	8	12	0.67	FAIR
Burge et al., 2003	Y	Y	Y	Y	N	N	Y	NA	Y	N	Y	NA	CD	Y	8	12	0.67	FAIR
Burge et al., 2008	Y	Y	Y	Y	N	N	Y	NA	Y	N	Y	NA	CD	Y	8	12	0.67	FAIR
Chang et al., 2014	Y	Y	CD	Y	N	N	N	NA	Y	N	Y	NA	Y	N	6	12	0.5	LOW
Conlon et al., 2019	Y	Y	Y	Y	N	N	N	NA	Y	N	Y	NA	CD	Y	7	12	0.58	FAIR
Forst et al., 2017	Y	Y	Y	Y	N	N	Y	NA	Y	N	Y	NA	CD	Y	8	12	0.67	FAIR
Ho et al., 2011	Y	Y	Y	Y	N	N	Y	NA	Y	N	Y	NA	CD	Y	8	12	0.67	FAIR
Hu et al., 2014	Y	Y	Y	Y	N	N	Y	NA	Y	N	Y	NA	CD	Y	8	12	0.67	FAIR
Hunt et al., 1998	Y	Y	Y	Y	N	N	Y	NA	Y	N	Y	NA	CD	Y	8	12	0.67	FAIR
Kao et al., 2018	Y	Y	Y	Y	N	N	Y	NA	Y	N	Y	NA	CD	Y	8	12	0.67	FAIR
Keating et al., 2008	Y	Y	Y	Y	N	N	Y	NA	Y	N	Y	NA	CD	Y	8	12	0.67	FAIR
Lackan et al., 2004	Y	Y	Y	Y	Y	N	Y	NA	Y	N	Y	NA	CD	Y	9	12	0.75	HIGH
Lavergne et al., 2011	Y	Y	Y	Y	N	N	Y	NA	Y	N	Y	NA	CD	Y	8	12	0.67	FAIR
Lin et al., 2013	Y	Y	Y	Y	N	N	Y	NA	Y	N	Y	NA	CD	Y	8	12	0.67	FAIR
McCarthy et al., 2003	Y	Y	Y	Y	N	N	Y	NA	Y	N	Y	NA	CD	Y	8	12	0.67	FAIR
Qureshi et al., 2019	Y	Y	CD	Y	N	N	Y	NA	Y	N	Y	NA	CD	Y	7	12	0.58	FAIR
Rosenwax et al., 2006	Y	Y	CD	Y	N	N	Y	NA	Y	N	Y	NA	CD	Y	7	12	0.58	FAIR
Sheffield et al., 2011	Y	Y	CD	Y	N	N	Y	NA	Y	N	Y	NA	CD	Y	7	12	0.58	FAIR
Shugarman et al., 2007	Y	Y	CD	Y	N	N	N	NA	Y	N	Y	NA	CD	Y	6	12	0.50	LOW
Shugarman et al., 2008	Y	Y	CD	Y	N	N	N	NA	Y	N	Y	NA	CD	Y	6	12	0.50	LOW
Soo et al., 2011	Y	Y	CD	Y	N	N	Y	NA	Y	N	Y	NA	CD	N	6	12	0.50	LOW
Walter et al., 2018	Y	Y	Y	Y	Y	N	Y	NA	Y	N	Y	NA	CD	Y	9	12	0.75	HIGH
Wang et al., 2016	Y	Y	CD	Y	N	N	Y	NA	Y	N	Y	NA	CD	Y	7	12	0.58	FAIR

Note: NHLBI= National Heart Lung and Blood Institute, Y=Yes, N=No, NA=Not Applicable, CD=Cannot Determine

Table S4. Characteristics summary of included studies.

**Author[#] /Year Country**	**Study period Age(years)**	**Study aim (EOL period)**	**Inclusion criteria (sample)**	**Cancer type/site (Classification)**	**Exposure variable/s**	**Outcome variable/s (time point/s)**	**Summary of findings Effect size (95% CIs)**	**Adjusted factor/s**	**Quality**
Bainbridge et al., 2015[33] Canada	2006 69–90+	Determine factors associated with LTC residents having an ED visit and dying in acute care (hospital or ED) (6 months)	Died of cancer in 2006; were in LTC in the final 6 months of life. Excluded if hospitalised for entire final 6 months of life (N=1196)	Lung Colorectal Prostate Breast Haematological Upper gastro-intestinal (ICD-9)	Rurality	ED visit (last 6 months of life) Death in acute care	61% visited ED in last 6 months (average 2.3 visits per person), 20% died in hospital Being younger and region of residence significantly increased the odds of an ED visit and/or hospital death (*p*<0.05)	Age, Gender, Income quintile, Cancer type, comorbidities	High
Barbera et al., 2006[36] Canada	2001 20+	Measure the proportion of ICU admissions, ER visits, chemotherapy (2 weeks)	Died of cancer in 2001 as identified in the Ontario Cancer Registry (OCR) (N=21,323)	Head and Neck Breast, Lung Prostate, Ovary Colorectal CNS, GU/gyne lymphoma/leukemia, melanoma/sarcoma (ICD-9)	Rural residence	ER visit (last 2 weeks of life) ICU visits (last 2 weeks of life) Chemotherapy (last 2 weeks of life)	27% visited ER and 5% visited ICU at least once in the last 2 weeks of life. Receiving a home care visit (last 6 months) or physician house call or palliative care assessment (last 2 weeks) was associated with decreased odds of ER visit, ICU visit, chemotherapy	Age, gender, regional, rural, Income quintile, cancer cause of death, comorbidities	High
Burge et al., 2003[37] Canada	1992–1997 <45–85+	To determine if greater family physician continuity of care for cancer patients during the end-of-	Died of cancer between 1992–1997; pathology report confirming cancer diagnosis; ≥3 ambulatory	Lung Colorectal Breast Prostate (ICD-9)	Urban/Rural residency	ED visits Family physician home visits, ambulatory visits, visits to	Patients experiencing low continuity (MMCI < 0.5) made 3.9 times more ED visits (rate ratio RR= 3.93; 95% CI= 3.57–4.34) than those experiencing high continuity	survival, death (year), sex, age, cancer type, PCP admission, specialty visits,	High

		life is associated with less ED utilisation (6 months)	visits to family physician in last 6 months of life (N=8,702)			a medical specialist, Days spent as a hospital inpatient	(MMCI > 0.8) and patients experiencing moderate continuity (MMCI =0.5–0.8) made twice as many ED visits (RR= 2.28; CI=2.15–2.42)	hospital days, death location, income quintile, total ambulatory visits	
Burge et al., 2008[34] Canada	1998–2003 18+	To examine the association between age and palliative care registration (6 months)	Died of cancer between 1998–2003; living in two district health authorities Nova Scotia, Canada (N=7,511)	Breast Lung Colorectal Gastrointestinal Prostate Genitourinary Gynaecologic Haematological (ICD-9) (ICD-10)	Urban/Rural indictor	PCP enrolment (by age) Predictors of palliative care enrolment (individual level/environmental factors)	Distance to the closest cancer centre was a significant issue among the elderly. Subjects residing <51 km from a tertiary cancer centre were eight times more likely to have been registered to a PCP than those living further away. Patients ≥85 years and over were 17 times more likely to be registered with a PCP if they lived ≤10km from the cancer centre compared with those who lived >50 km away	Distance to nearest cancer centre, death (year) age, sex, survival time, education, employment, race, urban/rural residency, income quintile, physician visit, oncology consult, PR, homecare services	Fair
Chang et al., 2014[35] Taiwan	2009–2011 18–64+	To examine the association between aggressiveness of EOL care and SES in working-age terminal cancer patients in Taiwan (1 month)	Died of cancer between 2009–2011 as identified in the Taiwan National Health Insurance (NHI) Research Database (N=32,800)	Germ cell tumours Prostate Lung Liver Pancreatic Hematologic (ICD-9)	Urbanisation level of residence	Chemotherapy, >1 ER visit, >1 hospital admission, >14 days of hospitalization, ICU admission, death in acute care hospital	More aggressive EOL care was performed in urban areas than rural areas and differed according to SES. High-SES terminal cancer patients were associated with less chemotherapy (p<0.001), fewer ER visits (p<.001), less ICU admission (p<.001), lower rates of dying in acute-care hospitals (p<.001) when compared with low-SES terminal cancer patients	Age, sex, cancer diagnosis, postdiagnosis survival time, disease severity, urbanization level of residence, geographic location, SES	Low

Conlon et al., 2019[31] Canada	2007–2012 All ages	To describe access to palliative care and associations with the use of potentially aggressive EOL care with reference to rurality and location of residence (last 30 days)	Died of any cancer cause of death at least 30 days after initial diagnosis during 2007–2012 (N=95,685)	Hematologic Prostate Breast Colorectal Lung	Rural/Urban residence	Chemotherapy (last 14 days), ICU admission, >1 ED visit,>1 hospitalization (last 30 days)	North/Rural residents had significantly decreased odds of receiving palliative care (AOR = 0.82, 95% CI: 0.76–0.88), more likely to receive a form of potentially aggressive EOL care, more likely to die in an acute care hospital	Age, sex, income quintile, rural/urban residence, diagnosis to death (time), cancer cause of death, death (year)	Fair
Forst et al., 2017[30] USA	2001–2012 18+	To evaluate the rates and correlates of hospice use among patients with MG and identify predictive factors for overall hospice enrolment and hospice LOS	Died of cancer (histology of anaplastic astrocytoma, glioblastoma or anaplastic oligodendroglioma) (N=12,437)	Malignant Glioma (MG)	Urban/Rural residence	Hospice enrolment prior to death LOS in hospice (continuous, >3 days, >7 days)	Odds of hospice enrolment were lower with increasing household income (OR0.893, 95% CI 0.85–0.939) and residing in a rural zip code (OR0.832, 95% CI 0.737–0.94). Higher odds of a short stay in hospice in younger, male patients residing in a rural area	Age, sex, race, marital status, extent of surgery, radiation treatment, income, urban/rural residence, education	High
Ho et al., 2011[44] Canada/ USA	1993–2004 20+	To evaluate the aggressiveness of EOL cancer care in Ontario, Canada and compare patterns with those observed in the US	Died of any cancer between 1993–2004 (N=227,161)	Breast Lung Colorectal Hematological Prostate (ICD-9)	Region of residence	Chemotherapy (14 days) >1 ED visit >1 hospitalisation (30 days) >1 ICU admission (30 days of death)	Age, male, region of residence and rurality were significant independent predictors of aggressive EOL care. Men were more likely to experience aggressive care (OR, 1.28; 95% CI, 1.25to 1.31) and rural-dwelling patients (OR, 1.34, 95% CI, 1.30–1.38)	Age, sex, income, region of residence, income, disease duration, comorbidity, cancer type	High
Hu et al., 2014 Canada	2006–2009 20+	To evaluate the aggressiveness of EOL care in Alberta, Canada,	Died of invasive colorectal cancer in Alberta between	Colon Rectum Rectosigmoid (ICD-O-3)	Region of residence	Chemotherapy (last 14 days of Life); >1 ER visit, >1	Living in a rural area were related to more aggressive care. Patients who lived in rural regions had 2.0–4.3	Age, sex, region of residence, death (year),	High

		for individuals who died of colorectal cancer (CRC) (30days)	2006–2009 (N=2,074)			hospitalization, ICU admission (last 30 days of life); dying in an acute care hospital	times the adjusted odds of having multiple ER visits in the last 30 days of life and3.7–12 times the odds of having >1 indicator of aggressive EOL care	cancer site/stage, disease duration, comorbidity, oncology services, radiotherapy, chemotherapy	
Hunt et al., 1998 Australi a	1999 <60–80+	To determine the extent of coverage by designated palliative care services of the population of terminally ill cancer patients in South Australia	Died with advanced malignancy in 1999 (N=3,086)	Upper digestive Colon/rectu m Liver, Lung Female Breast Prostate Lymphoma Multiple Myeloma Leukemia (ICD-9)	Place of residence	Designated hospice and palliative care services	Utilization of palliative services was lower among country residents than Adelaide residents (OR=0.57; 95% CI 0.48, 0.68)	Age at death, primary site of cancer (ICD-9 codes), country of birth, race, survival time from diagnosis, place of death, gender, SES	High
Kao et al., 2018 Taiwan	2010–2012 NR	To investigate the factors for ED during out-of-hours periods of palliative home care service among advanced cancer patients in Taiwan	Advanced cancer (any) patients enrolled in a hospice shared-care program in a community hospital receiving palliative home care (N=65)	Liver Lung Colorectal	Urbanisatio n level of residence	Emergency services used<br><br>ED admissions	Emergency services used in advanced cancer patients during out-of-hours periods of palliative home care was lower for individuals with an urban level of residence (OR:0.76; 95% CI 0.23–2.36) *p*=0.645	Age, sex, marital status, education, cancer type, morphine dose prior to ED admission, distressing condition	High
Keating et al., 2008	1992–1999 ≥65	To assess whether care before death, including the types of physicians	Died of stage3/4 breast cancer between 1992 and	Breast (SEER)	Residence	Hospice care<br><br>Duration of	No differences in hospice use by age, race/ethnicity, or marital status, but women in metropolitan areas were more	Age, race, ethnicity, marital status, history of other	High

USA		seen, number of out-patient visits and hospitalizations was associated with hospice use and the timing of enrolment	the end of 2001 (N=4,455)			enrolment<br><br>Late hospice enrolment (within 14 days of death)	likely than other women to use hospice care	cancer, cancer stage, death (date), income, comorbidity	
Lackan et al., 2004 USA	1991–1998 ≥67	To compare hospice use for Hispanics and non-Hispanic whites dying of cancer and examine hospice use over time and identify correlates of hospice use	Died of breast, colorectal, lung, or prostate cancer between 1991–1998 (N=34,336)	Breast Colorectal Lung Prostate (ICD-9)	Urban/rural residence	Hospice utilisation	Ethnicity, age at death and having a diagnosis of colorectal cancer were the only variables not associated with hospice use.	age, ethnicity, sex, marital status, education, death (year) tumour characteristics (size and stage), income, insurance	High
Lavergne et al., 2011 Canada	2000–2005 ≥20	To understand factors affecting access to PRT	Died of cancer between 2000–2005 in Nova Scotia, Canada (N=13,494)	Breast, Lung Colorectal Head and Neck Haematological Melanoma Pancreas Prostate (ICD-10)	Rural residence	PRT consultation (9 months)<br>Road travel time	Longer travel time may be a greater barrier for older people accessing palliative care programs	Age, sex, death (year), cancer site, survival time (months), deprivation, nursing home resident	High
Lin et al., 2013 Taiwan	2000–2006 ≥20	To investigate whether the urban-rural disparity widens in a country with a	Patients with cancer and received inpatient hospice care from	Lung, Hepatic Colon/rectal Head and Neck	Patient residence	Inpatient hospice care utilisation (patient-days)	The location of hospices played a major role of hospice utilization in rural areas	Age, gender, cancer diagnosis, monthly income, no. of	High

		hospital-based hospice system	2000–2006 (N=26,292)	Gastric, Breast Pancreatic Urinary Tract Prostate Haematological Oesophageal (ICD-9)		Location of first-time hospice care enrolment		hospices, no. of beds,	
McCarthy et al., 2003 USA	1973–1996 ≥66	To identify factors associated with hospice enrolment and length of stay in hospice among patients dying with lung or colorectal cancer	Died with primary diagnosis of lung or colorectal cancer between 1988–1998 (N=119,377)	Lung Colorectal (SEER)	Geographic area of residence	Time to hospice enrolment<br><br>Length of stay in hospice	Patients residing in rural communities enrolled in hospice later, had 35% lower enrolment and had longer stays (colorectal cancer) than did those in urban communities	Age, sex, race, marital status, median household income, SEER tumour stage at diagnosis	High
Qureshi et al., 2019 Canada	2010–2012 18+	To investigate the association between early vs. late palliative care and acute-hospital use (last 2 weeks of life)	Died with causes; (terminal illness, organ failure, frailty, sudden death) between 2010–2012 (N=230,921)	All cancer types/codes (ICD-10)	Rurality	Use of acute-care and community services (last 2 weeks)	Early palliative care is associated with reduced acute-hospital use and urban residents had lower odds of using acute care settings in the last 2 weeks of life	Age, sex, income comorbidities, rurality, place of death, time to first palliative care initiation	Fair
Rosenwax et al., 2006 Australia	2000–2002 0–85+	To quantify the use of palliative care in people dying of cancer vs. selected non-cancer conditions (12 months)	Died of cancer or selected non-cancer condition deemed amenable to palliative care (N=7399)	All cancer types/codes (ICD-10)	Geographic remoteness	Use of SPCS (hospital based and community based) (12 months), Predictors of receiving SPCS	Cancer patients were less likely to receive SPCS if single/widow, >85 years or lived outside a major city. 2/3 cancer patients received SPCS compared to 1/10 of non-cancer patients	Age, gender, marital status, aboriginality, IRSD, ARIA, geographical remoteness	Fair
Sheffield et al.,	1992–2006 <70–85+	To examine hospice use and aggressive-ness of	Died of pancreatic cancer between 1992–2006;	Pancreatic (ICD-9)	Geographic area of residence	Hospice use, hospice enrolment ≥4	Racial/ethnic minorities and patients in rural areas were less likely to use hospice care.	Age, sex, race/ethnicity,	Fair

2011 USA		care for pancreatic cancer patients at the end of life (≤4 weeks)	enrolled in Medicare parts A and B without any HMO enrolment for 12 months before and 3 months after diagnosis (N=22,818)			weeks before death, aggressiveness of care; chemotherapy, acute care hospitalization, ICU admission (last 1 month)	Early enrolment in hospice was more common among females, Hispanics and whites, unmarried patients and patients living in rural areas	marital status, geographic area of residence (urban, rural), income, education, comorbidity, tumour location, cause of death	
Shugar man et al., 2007 USA	1996–1999 ≥68	To examine age and gender differences in service use and expenditures for colorectal cancer decedents (12 months)	Died with colorectal cancer diagnosis between 1996–1999 eligible for fee-for-service Medicare (N=6,657)	Colorectal (ICD-9)	Geographic region of residence	Utilisation and expenditure for: inpatient, outpatient, physician, skilled nursing facility, home health, hospice (last 36 months)	Most of the gender differences in average Medicare expenditures were explained by gender differences in age and the lower average expenditures on older decedents with colorectal cancer	Age, gender, race, Medicaid enrolment, area income, comorbidities, illness duration, service utilization, rural/urban residence, physician supply SNF bed supply	Low
Shugar man et al., 2008 USA	1996–1999 ≥68	To examine age and gender differences in service utilisation and expenditures for lung cancer decedents (12 months)	Died with lung cancer diagnosis between 1996–1999 eligible for fee-for-service Medicare (N=241, 047)	Lung (ICD-9)	Geographic region of residence	Utilisation and expenditure for: inpatient, outpatient, physician, skilled nursing facility, home health, hospice	Gender disparities in expenditures are generally small at the end of life for lung cancer decedents, particularly among the older cohorts	Age, gender, race, Medicaid enrolment, area income, comorbidities, metastatic disease, illness duration, service	Low

						(last 36 months)		utilization, rural/urban residence, physician supply, SNF bed supply	
Soo et al., 2011 Canada	1986–2005 <65–85+	To examine the accessibility RT with palliative intent in the treatment of prostate cancer in BC, Canada	Died between 1990–2005; diagnosed with invasive prostate cancer 1986–1999; received RT between 1986–2005. Excluded cases classified as benign, borderline, in situ	Prostate (CAIS)	Citizen residence	Overall RT utilization rate, PRT utilization rate (PUR), Referred RT utilization rate, Multiple course rate (MCR)	Access to and utilization of RT is lower in remote geographical regions and higher in urban regions where a cancer care facility is close in proximity suggesting the presence of geographical barrier affecting access to health care and services	Age at diagnosis, Age at death	Fair
Walter et al., 2018 Germany	2009–2013 NR	To assess rural-urban differences in healthcare utilization and supportive care at the EOL in German lung cancer patients (30 days)	Died before 2013 but survived for >30 days after diagnosis; diagnosed with incident lung cancer 2009–2012 (N=12,929)	Lung (ICD-10)	Residential area	Hospitalisations, >14 days hospital admission, doctor/home visits, palliative care, prescription antidepressant treatment, pain medication (30 days), chemotherapy, therapeutic puncturing (2 weeks)	The likelihood of >14 hospital days in the last 30 days was significantly higher in rural districts than in remote rural districts (1.27 [1.05, 1.52], p=0.0003). The number of visits to the GP in the last 30 days of life was significantly lower in urban districts than in remote rural districts (β =−0.19 [−0.32, −0.06], p=<0.0001)	sex, age, comorbidities, presence of tumours, type of metastases, survival after diagnosis, anti-cancer treatment	High

Wang et al., 2016 USA	2006–2011 ≥66.5	To examine contemporary trends in end-of-life cancer care and geographic variation of end-of-life care aggressiveness (18 months)	Died of cancer between 2006–2011; diagnosed with cancer in 2004–2011 (breast, prostate, lung, colorectal, pancreas, liver, kidney, hematologic cancer or melanoma) (N=132,051)	Breast Colorectal Lung Prostate (ICD-9)	Metropolitan status of residence	Chemotherapy (14 days), >1 ED visit, >1 hospitalization, ≥1 ICU admission (30 days); in-hospital death; hospice enrolment (<3 days before death), EOL care	Regional variation of EOL cancer care was substantial with no evidence of decreased variation over time. Decedents who were younger, male, non-white, Hispanic, married, resided in non-metropolitan areas or areas with higher proportions with more than high school education were more likely to have aggressive care	Age, race, gender, death (year), marital status, SEER registry, metro status of residence, income, education, disability status, outpatient clinic visits, tumour characteristics	Fair

Note: [#]—surname of the first author et al. is captured in this table, CS=Cohort Study, CSS=Cross-sectional study, CCS=Case-Control study, LTC=Long-term Care, COD=Cause of death, PCCF=Postal Code Conversion File, HRM=Halifax regional municipality, PCP=Palliative Care Program, ER=Emergency Room, RUCA=Rural-Urban Commuting Area, SPCS=Specialist Palliative Care Services, ARIA=Accessibility Remoteness Index of Australia), SNF=Skilled Nursing Facility, LYOL=Last Year of Life, RT=Radiation Therapy, PRT=Palliative Radiation Therapy, HSDA=Health Service Delivery Area, EOL=End Of Life, ICD=International Classification of Disease, NR=Not Reported, SEER= Surveillance, Epidemiology and End Results Medicare data registry, CAIS=BC Cancer Agency Information System.

Table S5. Summary of geographical urban-rural measures and influence on end-of-life cancer care health service use outcomes.

Urban-rural measure	Definition	Author#	Database used	Health service/s used	Influence on end-of-life cancer care health service use
Region of residence	Rural areas are defined as census sub-divisions and included small towns and municipalities with a population <10,000 that are outside the commuting zones of urban centres	Bainbridge et al., 2015[33]	2006 Canadian Census of the Populations and the Registered Persons Database (RPD)	ED visit<br>Acute care	Rural location were not strong predictors of the acute care outcomes. However, certain comorbidities, being younger and region of residence significantly increased the odds of an ED visit and/or hospital death (p<0.05).
	Rural included rural/small towns and municipalities outside of larger urban centres >10,000 population and constructed from census subdivisions	Barbera et al., 2006[36]	Postal code data from Registered Persons Database (RPD)	ER visit<br>ICU visits<br>Chemotherapy	Rurality was associated with an increased odds of ER visit (AOR:1.116 95% CI 1.019–1.22) and decreased odds of an ICU visit (AOR:0.651 95% CI 0.52–0.80) and chemotherapy (AOR:0.99 95% CI 0.79–1.25)
	Rural residence as communities with <10,000 people and not located near commuting zones of metropolitan areas. Urban residence all other areas	Conlon et al., 2019[31]	2006 Canadian Census of the Populations and the Registered Persons Database (RPD)	Chemotherapy<br>ICU admission<br>ED visit<br>Hospitalization	Rural residents had significantly decreased odds of receiving palliative care (AOR: 0.82, 95% CI: 0.76–0.88), more likely to receive a form of potentially aggressive EOL care, more likely to die in an acute care hospital
	Rural residence defined as communities with a population size of <10,000	Ho et al., 2011[44]	2006 Canadian Census of the Populations and the Registered Persons Database (RPD)	Chemotherapy<br>ER visit<br>Hospitalization<br>ICU admission	Region of residence and rurality were significant independent predictors of aggressive EOL care. Rural-dwelling patients were more likely to experience aggressive care (OR: 1.34, 95% CI, 1.30–1.38)
Urban/Rural residency	Geographic indicator derived from PCCF and 1996 census data	Burge et al., 2003[37]	The postal code conversion file (PCCF) and 1996 Canadian Census data	ED visits<br>Physician home visits<br>Ambulatory visits<br>Medical specialist visit<br>Hospital inpatient	Rural residency was associated with an increased odds of ED visits by continuity score and patient characteristics (AOR:1.09 95% CI 1.02–1.16)
Urban/Rural indictor	Urban areas defined as regions ≥1000 population concentration and population density ≥ 400 persons per km². All other areas are considered rural	Burge et al., 2008[34]	Postal code and Census data from Statistics Canada 2001	Chemotherapy<br>ER visit<br>Hospital admission<br>ICU admission	Residents in rural regions compared with urban regions were less likely to be registered with Palliative Care Program. Distance to closest cancer centre also had a major impact on registration.

Urban/Rural residence	Indicated by residence in a rural zip code	Forst et al., 2017[30]	SEER-Medicare database	Hospice enrolment<br>LOS in hospice	The odds of hospice enrolment were lower with increasing household income (OR:0.893, 95% CI 0.85–0.939) and residing in a rural zip code (OR0.832, 95% CI 0.737–0.94)
Region of residence	Five geographic areas corresponding to provincial health care zones based on population size: urban/suburban in population size and density, suburban, rural, remote regions	Hu et al., 2014	Alberta Cancer Registry	Chemotherapy<br>ER visit<br>Hospitalization<br>ICU admission	Patients who lived in rural regions had 2.0–4.3 times the adjusted odds of having multiple ER visits in the last 30 days of life and3.7–12 times the odds of having >1 indicator of aggressive EOL care
Place of residence	Country residents (rural) defined as living outside of Adelaide city	Hunt et al., 1998	Postcode data from the Adelaide Statistical Division	Hospice<br>Palliative care services	Utilization of palliative services was lower among country residents than Adelaide residents (OR=0.57; 95% CI 0.48, 0.68)
Urbanisation level of residence	Location of residence classified into urban versus suburban and rural levels of urbanization	Kao et al., 2018	Medical records from a community hospital in southern Taiwan	Emergency services<br>ED admissions	Emergency services used in advanced cancer patients during out-of-hours periods of palliative home care was lower for individuals with an urban level of residence (OR: 0.76; 95% CI 0.23–2.36) p=0.645
Residence in a metropolitan county	Defined as non-metropolitan and metropolitan county	Keating et al., 2008	SEER-Medicare database	Hospice	No differences in hospice use by age, race/ethnicity or marital status, but women in metropolitan areas were more likely than other women to use hospice care
Urban/Rural residence	Urban areas were designated as counties with a population greater than 250,000 in or adjacent to large metropolitan areas	Lackan et al., 2004	SEER-Medicare database	Hospice	Odds of hospice use was higher among individuals living in an urban area of residence
Rural residence	Rural residence was defined by 2nd character of postal code of residence	Lavergne et al., 2011	Postal code and Census data from Statistics Canada	PRT	Living in a rural area was a predictor of lower rates of PRT consultation and treatment
Patient residences	Residences defined as the location of physician clinics patients most frequently sought medical help for upper respiratory tract infection in five years	Lin et al., 2013	National Health Insurance Research Database (NHIRD) Taiwan	Hospice	The location of hospices played a major role of hospice utilization in rural areas
Geographic area of residence	Geographic areas were defined as urban or rural based on SEER database	McCarthy et al., 2003	National Cancer Institute's SEER Program, USA	Specialist care services<br>Hospital admissions<br>SPCS access	The rate of hospice enrolment among rural patients was approximately 35% lower than that of urban patients for both primary

					cancer sites
Rurality	Rurality was categorised based on postal code data	Qureshi et al., 2019	Statistics Canada Census data	Use of SPCS (hospital based and community based), Predictors of receiving SPCS	Early palliative care is associated with reduced acute-hospital use and urban residents had lower odds of using acute care settings in the last 2 weeks of life
Geographical remoteness	Geographical remoteness was recorded as either a major city of Australia, inner regional Australia, outer regional Australia, remote Australia, very remote Australia and migratory	Rosenwax et al., 2006	2001 Census of Population and Housing and the Accessibility Remoteness Index of Australia (ARIA)	Hospice use, hospice enrolment ≥4 weeks before death, aggressiveness of care; chemotherapy, acute care hospitalization, ICU admission	Cancer patients were less likely to receive SPCS if single/widow, >85 years or lived outside a major city
Geographic area of residence	Geographic areas of residence defined as urban or rural	Sheffield et al., 2011	National Cancer Institute's SEER tumour registry	EOL patterns of care; Hospice discharge, In-hospital death	Patients in rural areas were less likely to use hospice care, early enrolment was more common among females, Hispanics and whites, unmarried patients and patients living in rural areas
County of residence	County of residence defined as metropolitan, small urban or rural	Shugarman et al., 2007	Linked Area resource file and Medicare Denominator file	Inpatient, outpatient, physician, skilled nursing facility, home health, hospice	Patients in rural county of residence were less likely to use hospice services than their urban counterparts (0.64 [0.55–0.75], *p*<0.05)
	County of residence defined as metropolitan, small urban or rural	Shugarman et al., 2008	Linked Area resource file and Medicare Denominator file Influence Codes	Overall RT utilization rate, PRT utilization rate, Referred RT utilization rate, Multiple course rate	Patients in rural county of residence were less likely to use hospice services than their urban counterparts (0.66 [0.59–0.73], *p*<0.05)
Citizen residence	Residence was defined by British Columbia 16 HSDAs (urban, suburban, remote rural)	Soo et al., 2011	BC Cancer Registry and BC Cancer	Hospitalisations, doctor/home visits, palliative care,	Access to and utilization of RT is lower in remote geographical regions and higher in

			Agency Information System (CAIS)	prescription antidepressant treatment, pain medication, chemotherapy, therapeutic puncturing	urban regions where a cancer care facility is close in proximity
Residential area	Residential area based off the zip code of last place of residence data. Classified as (major city, urban districts, rural districts, remote rural districts)	Walter et al., 2018	Scientific Institute of AOK SHI fund	Chemotherapy, ED visit, hospitalization, ICU admission; in-hospital death; hospice enrolment, EOL care	The likelihood of >14 hospital days in the last 30 days was significantly higher in rural districts than in remote rural districts (1.27 [1.05, 1.52], p=0.0003). The number of visits to the GP in the last 30 days of life was significantly lower in urban districts than in remote rural districts (β =−0.19 [−0.32, −0.06], p=<0.0001)
Metropolitan status of residence	Metropolitan status of residence derived defined as metropolitan and non-metropolitan areas	Wang et al., 2016	SEER-Medicare database	ED visit<br>Death in acute care	Regional variation of EOL cancer care was substantial with no evidence of decreased variation over time. Decedents who were younger, male, non-white, Hispanic, married, resided in non-metropolitan areas were more likely to have aggressive care

Note: #surname of the first author et al. is captured in this table, PCCF=Postal Code Conversion File, RPD=Registered Persons Database, ER=Emergency Room, ED=Emergency Department, SPCS=Specialist Palliative Care Services, ARIA=Accessibility Remoteness Index of Australia), EOL=End Of Life, LYOL=Last Year of Life, SEER= Surveillance, Epidemiology and End Results Medicare data registry, CAIS=BC Cancer Agency Information System.
